# Spatiotemporal distribution of ground-level ozone in China at a city level

**DOI:** 10.1038/s41598-020-64111-3

**Published:** 2020-04-29

**Authors:** Guangfei Yang, Yuhong Liu, Xianneng Li

**Affiliations:** Institute of Systems Engineering, Dalian University of Technology, Dalian, China

**Keywords:** Environmental impact, Sustainability

## Abstract

In recent years, ozone (O_3_) pollution in China has shown a worsening trend. Due to the vast territory of China, O_3_ pollution is a widespread and complex problem. It is vital to understand the current spatiotemporal distribution of O_3_ pollution in China. In this study, we collected hourly data on O_3_ concentrations in 338 cities from January 1, 2016, to February 28, 2019, to analyze O_3_ pollution in China from a spatiotemporal perspective. The spatial analysis showed that the O_3_ concentrations exceeded the limit in seven geographical regions of China to some extent, with more serious pollution in North, East, and Central China. The O_3_ concentrations in the eastern areas were usually higher than those in the western areas. The temporal analysis showed seasonal variations in O_3_ concentration, with the highest O_3_ concentration in the summer and the lowest in the winter. The weekend effect, which occurs in other countries (such as the USA), was found only in some cities in China. We also found that the highest O_3_ concentration usually occurred in the afternoon and the lowest was in the early morning. The comprehensive analysis in this paper could improve our understanding of the severity of O_3_ pollution in China.

## Introduction

Air pollution has rapidly increased over the last few decades due to urbanization and industrialization, and this increase has attracted attention around the world, including in China. In 2012, the National Ambient Air Quality Standards (NAAQS) (GB 3095-2012) was published by the Chinese Ministry of Environmental Protection, which identified six environmental pollutants: sulfur dioxide (SO_2_), nitrogen dioxide (NO_2_), carbon monoxide (CO), ozone (O_3)_, and particulate matter (PM_2.5_ and PM_10_)^1–3^. Several air pollution control policies and programs have been established by the Chinese government^4–6^. However, there is still a gap between China’s ambient air quality and the air quality guidelines (AQG) of the World Health Organization (WHO)^7^. In recent years, although the concentrations of most pollutants, including NO_2_, SO_2_, particulate matter, and CO, decreased in the period of 2013–2016, O_3_ concentrations have increased by 10.79%^8^. O_3_ has become a secondary pollutant after PM_2.5_, which introduces new challenges to pollution control^9^.

In the past few years, many studies have investigated the impact, formation, and sources of O_3_ pollution. For instance, Khaniabadi *et al*.^10^ found that inhaling high concentrations of O_3_ or exposure to O_3_-polluted environments for a long period of time had a negative impact on health. Inhaling high concentrations of O_3_ can increase the risk of cardiovascular and respiratory diseases, which contribute to the overall mortality rate. Huang *et al*.^11^ found that ambient O_3_ exposure was related to the tremendous disease burden of chronic obstructive pulmonary disease in Ningbo, China, and the elderly comprised a more susceptible population. Existing evidence also reveals the adverse effects of O_3_ on mental health^12^. O_3_ pollution will not only have a negative impact on human health^13^ but also have a variety of adverse effects on plants, such as declines in crop yields and quality^14–16^. Because O_3_ has a negative impact on the transfer of nitrogen to grain, O_3_ pollution will reduce the fertilizer efficiency of wheat^17^, leading to the inhibition of net photosynthesis of wheat^18^. Additionally, O_3_ is a secondary pollutant that is formed by other pollutants through reactions^19–21^. Therefore, the formation of O_3_ pollution is affected by many factors^22–24^. Studies have shown that volatile organic compounds (VOCs) and NO_x_ are key precursors of O_3_ formation^23^. Aromatic hydrocarbons and olefins are considered the main contributors to O_3_ formation in many cities or regions in China. Ethylene, trans-pentene, propene, and BTEX (benzene, ethylbenzene, toluene, m-, p-, and o-xylene), as well as warm weather and low wind speeds, are also major contributors to O_3_ formation^25^_._ Given that China is currently plagued by complex O_3_ pollution problems, understanding the spatiotemporal pattern of O_3_ pollution in China is of great significance for conducting environmental epidemiological studies and drafting appropriate regional O_3_ pollution control strategies.

Some scholars have launched investigations on the spatiotemporal pattern of O_3_. In Nanjing, a unimodal peak was observed with the highest O_3_ levels occurring from 14:00 to 15:00, and the O_3_ concentration reached its maximum and minimum levels in the summer and winter, respectively^3^. Wang *et al*.^26^ studied the ground-level O_3_ concentrations of 6 major Chinese cities located on both sides of the Heihe-Tengchong line, and they found that ground-level O_3_ concentrations exhibited monthly variability, peaking in summer and reaching the lowest levels in winter. The diurnal cycle reached a minimum in the morning and peaked in the afternoon. Some research has found that the O_3_ distribution pattern is also related to terrain features^9,27^.

As previously mentioned, most of the studies on O_3_ spatiotemporal patterns are carried out with a short time scale and low spatial resolution and generally focus on a specific city or a limited spatial region. To the best of our knowledge, there has been a lack of research on the spatiotemporal pattern of O_3_ in China using a higher spatial resolution and long time-series datasets. Recently, China established a large-scale ground real-time air quality monitoring network, which provides data we can use to conduct research on the spatiotemporal distribution pattern of O_3_ pollution nationwide.

In brief, this research makes the following contributions. First, we obtained the O_3_ concentration data of 338 cities across China for more than three years, covering 1-Jan-2016 to 28-Feb-2019. In terms of spatial perspective, we investigated the O_3_ concentrations in seven major geographic regions and three major urban agglomerations to conduct a more in-depth analysis and discussion. In terms of temporal perspective, we studied the annual, seasonal, monthly, weekly, daily, and diurnal and nocturnal variations in the O_3_ concentrations. Second, the reasons for different patterns in different regions were briefly analyzed. The research results from this large dataset can not only help us elaborate on the spatiotemporal distribution pattern of O_3_ concentration in China with a better spatiotemporal resolution and increase public awareness of the current O_3_ pollution situation in China but also assist the relevant departments in formulating more targeted O_3_ pollution prevention and control policies to meet the NAAQS and even the AQG standards in the future.

## Results and Discussion

The NAAQS and the WHO set concentration limits for the maximum daily 8-hour average (MDA8) O_3_ concentration. Two levels of limits are specified in the NAAQS (Grade 1 and Grade 2), and three levels of limits are specified in the WHO standard (AQG, Interim target 1 and High level) (see Table 1).

**Table 1:** The O_3_ concentration limits of the NAAQS in China and the WHO (unit: μg/m^3^).

NAAQS		Who		
Grade 1	Grade 2	Air Quality Guidelines (AQG)	Interim target 1	High level
100	160	100	160	240

### Spatial distribution of O_3_ in China

**Figure 1:**
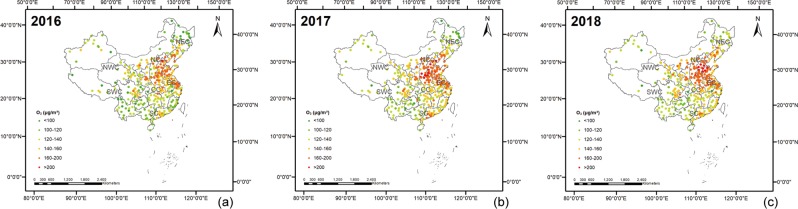
The spatial distribution of the 90th percentile of the maximum daily 8-hour average of the urban O_3_ concentration in 338 cities in China in 2016 (**a**), 2017 (**b**), and 2018 (**c**). The maps were generated in ArcGIS10.2, URL: http://www.esrichina-bj.cn/softwareproduct/ArcGIS/.

Figure 1 shows the spatial distributions of the O_3_ concentrations in 338 cities in China in 2016–2018. The regions with the most O_3_ pollution were mainly concentrated in North China and Central China, especially in the Beijing-Tianjin-Hebei region (BTH) region. In addition, the O_3_ pollution in the Chengdu-Chongqing and the Pearl River Delta region (PRD) regions was significantly higher than that of their neighboring regions. O_3_ pollution in China has shown a trend of outward expansion. As shown in Table 2, based on the statistical results of the 90th percentile of the maximum daily 8-hour average urban O_3_ concentration, the top 10 cities with severe O_3_ pollution are mainly located in North China, Central China and the East China.

**Table 2:** The top 10 cities with the highest 90th percentile of the maximum daily 8-hour average urban O_3_ concentration in 2016-2018 in China.

2016	2017	2018
1. Beijing (Beijing Municipality) 2. Tai’an (Shandong Province) 3. Dezhou (Shandong Province) 4. Hengshui (Hebei Province) 5. Dongying (Shandong Province) 6. Wuxi (Jiangsu Province) 7. Jinzhou (Liaoning Province) 8. Jinan (Shandong Province) 9. Panjin (Liaoning Province) 10. Heze (Shandong Province)	1. Linfen (Shanxi Province) 2. Baoding (Hebei Province) 3. Jincheng (Shanxi Province) 4. Anyang (Henan Province) 5. Jiaozuo (Henan Province) 6. Tai’an (Shandong Province) 7. Xingtai (Hebei Province) 8. Langfang (Hebei Province) 9. Tangshan (Hebei Province) 10. Luoyang (Henan Province)	1. Baoding (Hebei Province) 2. Jinan (Shandong Province) 3. Liaocheng (Shandong Province) 4. Binzhou (Shandong Province) 5. Jincheng (Shanxi Province) 6. Dezhou (Shandong Province) 7. Shijiazhuang (Hebei Province) 8. Xingtai (Hebei Province) 9. Cangzhou (Hebei Province) 10. Handan (Hebei Province)

**Figure 2:**
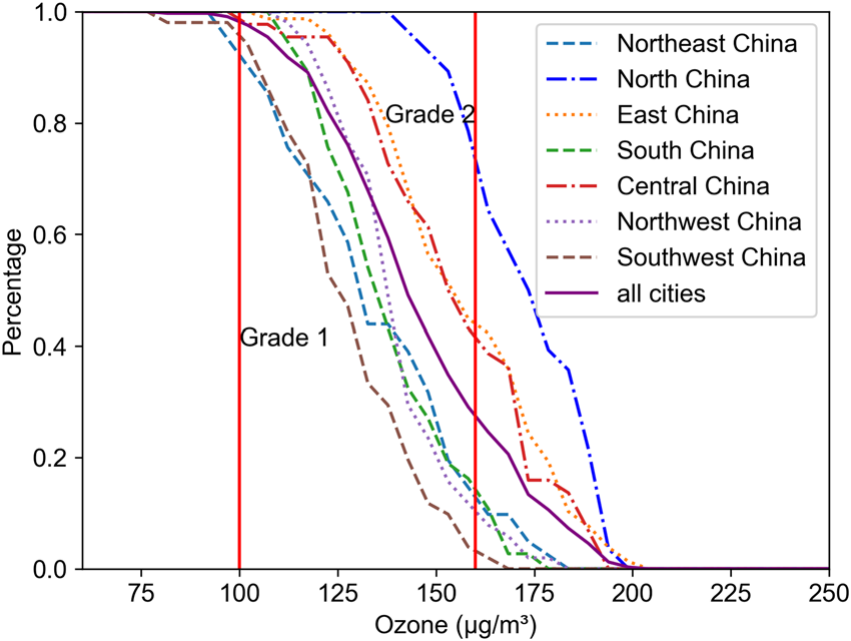
The over-standard rate of O_3_ concentration in 338 Chinese cities. The results of the seven geographical regions are also displayed.

Fig. 2 displays the over-standard rate of the O_3_ concentration in seven geographical regions in China. None of the cities in North China met the AQG or Grade 1 limit, and nearly 70% exceeded the Grade 2 limit. In East and Central China, nearly 40% of urban O_3_ concentrations exceeded the Grade 2 limit. The O_3_ pollution in other regions was not so prominent; however, there were still considerable gaps from the AQG standard.

O_3_ is a secondary pollutant, which is generally formed in the atmosphere through photochemical pathways of NOx and volatile organic compounds (VOCs)^28–32^. Most of the NO_x_ and VOCs come from heavy industries, such as coal-fired power plants, the steel industry, and the cement industry. Some studies found that the local photochemical reaction process has made an important contribution to the formation of O_3_^33^, including the consumption of NO_2_ during the photochemical reaction process^23^, which has been observed in regions such as North China and Yangtze River Delta (YRD) region^34,35^.

Additionally, PM pollution control in these regions has achieved certain results, and the reduction in haze has led to increased visibility, which in turn, has promoted the process of photochemical reactions and promoted the formation of O_3_ pollution. It is worth noting that some cities in western China, where industrial activities are infrequent, sometimes have high concentrations of O_3_. In these high-altitude regions, the increase in O_3_ concentration may be related to the transport of O_3_ from the stratosphere to the troposphere^36^. In addition, meteorological environments with a high ultraviolet intensity and low humidity are conducive to O_3_ formation. In general, the formation of O_3_ pollution is affected by many factors, including prerequisite pollutant concentrations and meteorological conditions.

### Annual variation in O_3_ in China

**Figure 3:**
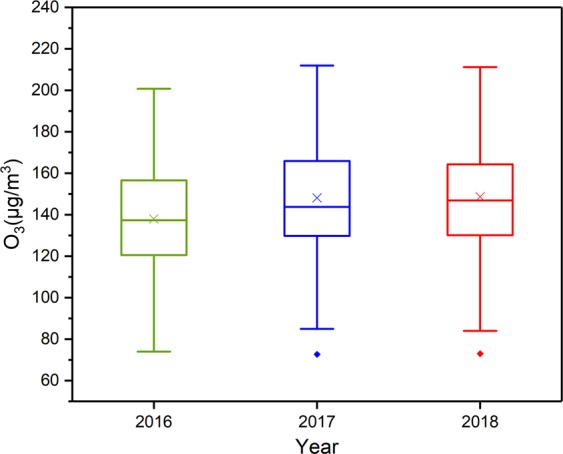
The box plots of the annual O_3_ concentrations of 338 cities in China in 2016–2018.

**Figure 4:**
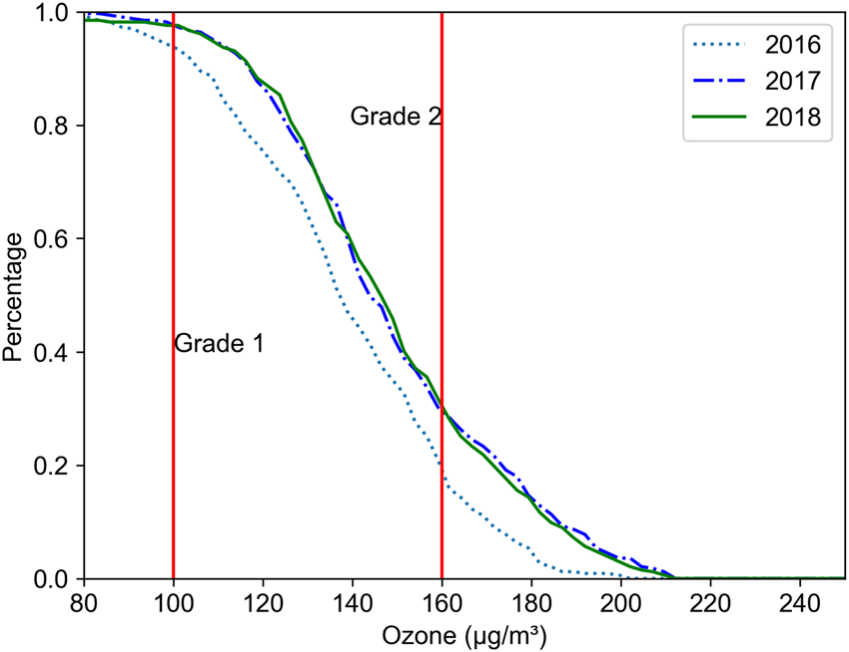
The annual over-standard rate of the O_3_ concentrations in 338 Chinese cities in 2016-2018.

Figure 3 shows the change in ozone concentration in all cities in China in 2016-2018. The top and bottom whiskers extend from the hinges to the largest values by no more than 1.5* IQR (interquartile range). The upper and lower bounds of the box represent the 75th and 25th quartiles, respectively. The line in the middle of the box represents the median. The cross points indicate the mean values, and the square points outside the whisker indicate outliers. From 2016 to 2018, the O_3_ concentration showed an upward trend, and the ozone levels were roughly the same in 2017 and 2018. Figure 1 shows that the scope of heavy O_3_ pollution has gradually expanded. This phenomenon is also depicted in Fig. 4. In 2016, more than 95% of cities failed to meet the Grade 1 standard, and nearly 20% failed to meet the Grade 2 standard. In 2017 and 2018, these values increased to 99% and 30%, respectively.

### Seasonal variation in O_3_ in China

**Figure 5:**
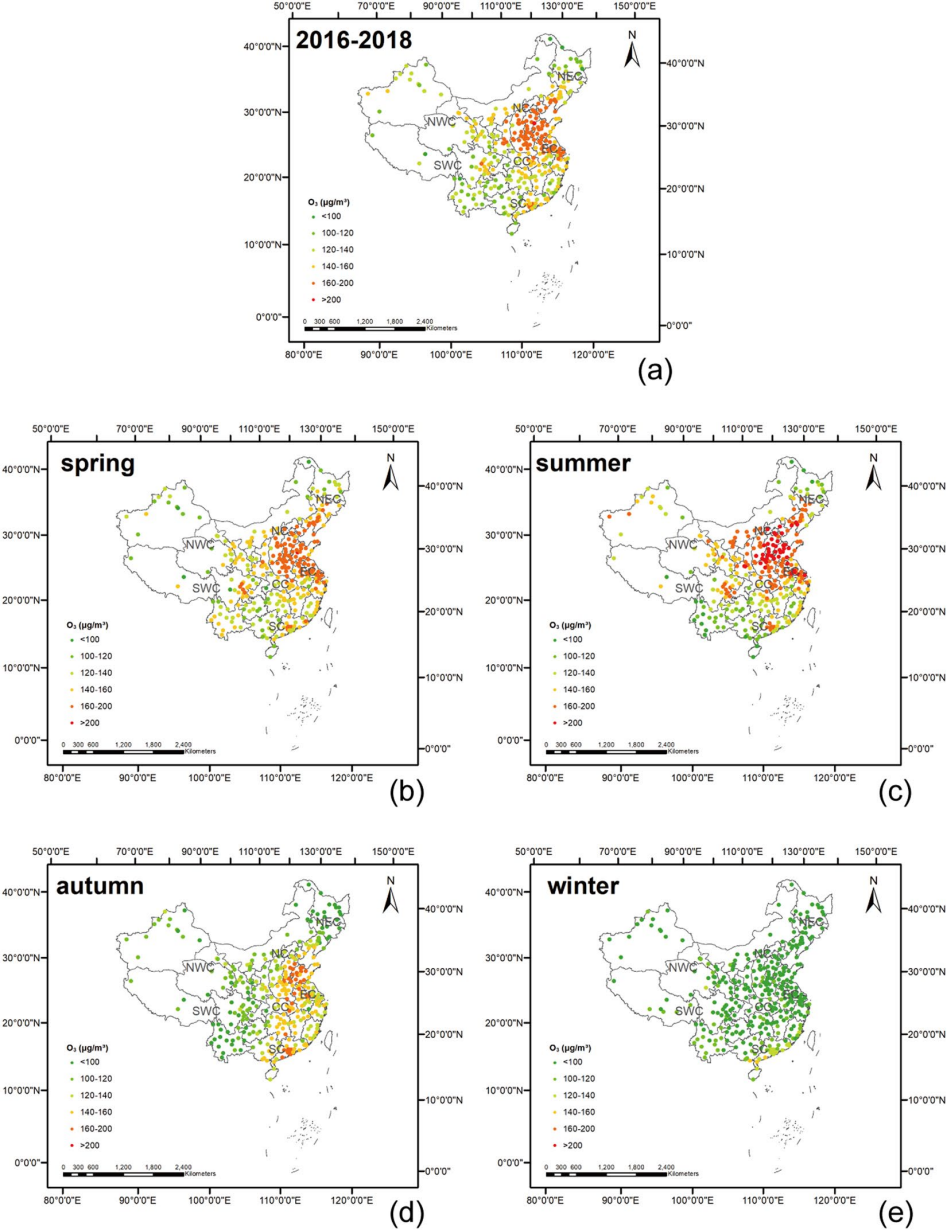
The average O_3_ concentrations of the 338 cities of China during 2016, 2017 and 2018 (**a**) and during spring (**b**), summer (**c**), autumn (**d**), and winter (**e**). The maps were generated in ArcGIS10.2, URL: http://www.esrichina-bj.cn/softwareproduct/ArcGIS/.

The distributions of O_3_ in different seasons were heterogeneous, exhibiting significant seasonal variations. In general, O_3_ pollution in the summer is significantly higher than that in winter (Fig. 5). Because the photochemical reaction process is affected by meteorological conditions such as light and temperature, the meteorological conditions in summer are more suitable for photochemical reactions. In contrast, the UV intensity in winter is low, and the photochemical reaction is not enough to form heavy O_3_ pollution.

Seasonality is also reflected in spatial variation. In the spring and summer, O_3_ pollution is highest in North, East, and Central China. In autumn, O_3_ pollution gradually shifts to the south. In winter, national O_3_ pollution is relatively mild, and only a small part of South China suffers from O_3_ pollution. Overall, the problem of O_3_ pollution in the eastern areas is more serious than that in the western areas. The seasonality of O_3_ concentration changes in the BTH region and the YRD region is relatively high. However, the seasonality of O_3_ concentration changes in the PRD region is not as obvious. In the BTH region and the YRD region, the maximum and minimum O_3_ concentrations were observed in the summer and winter, respectively. In the PRD region, the maximum O_3_ concentration was observed in autumn, and the minimum was observed in winter.

The formation of O_3_ pollution varies based on factors such as the overall NO_x_ and VOC emissions^37–39^, topography^40^, and atmospheric circulation in the region^31,41^. Evidence suggests that the high O_3_ pollution in the BTH region may be related to the emissions of precursor pollutants and the transportation of VOCs in neighboring provinces^42,43^. In the YRD region, the high temperatures in summer and the lower humidity can easily induce O_3_ pollution. The O_3_ concentration in the PRD region throughout the year is close and at a high level because the temperature throughout the year is similar and the annual average temperature exceeds 20°C in this region.

### Monthly variation in O_3_ in China

**Figure 6:**
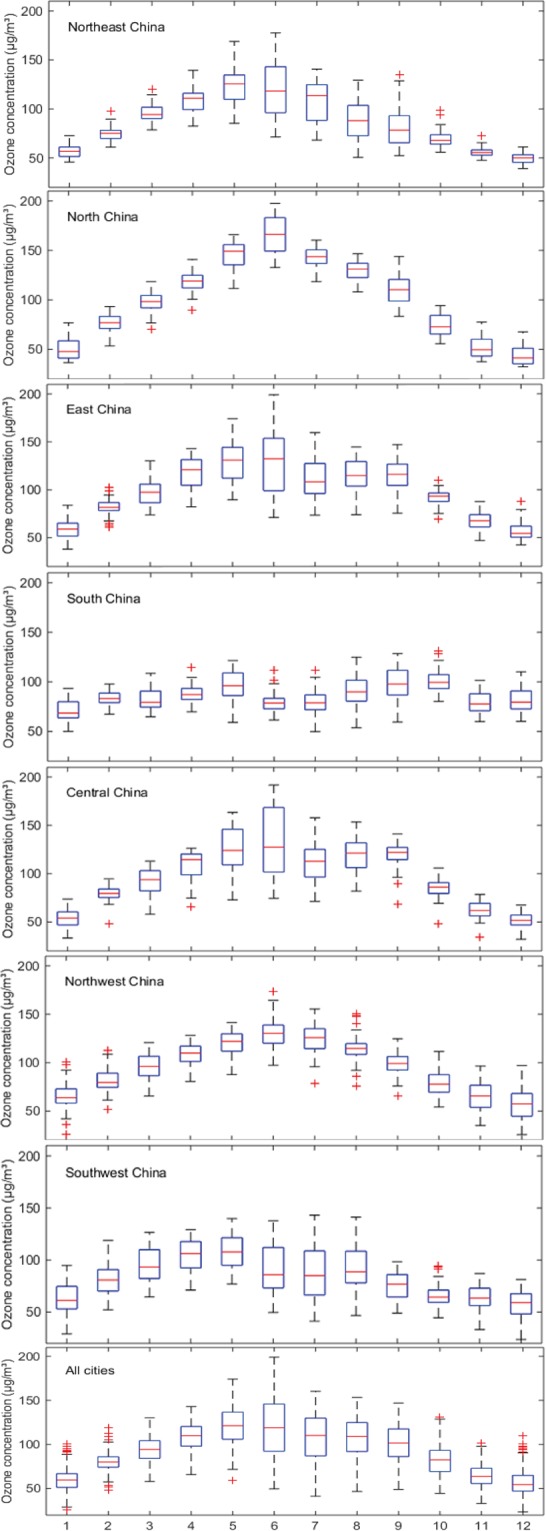
Monthly variation in the maximum daily 8-hour average concentration of O_3_ in seven geographical regions and in all cities during 2016-2018.

Figure 6 illustrates the highest maximum, upper-quartile, median, lower-quartile, and minimum values of the monthly variations in O_3_ concentration from January to December in the seven regions and the total for all cities. The data confirm that the O_3_ concentration changes periodically depending on the month. Except for South and Southwest China, the trends in the O_3_ concentration variations in other regions are consistent with the national trend, showing an inverted V-shaped curve. The O_3_ concentration gradually increases from January to June, reaching the highest value in June, and then gradually decreases from June to December. The trend of O_3_ concentration variations in South China and Southwest China is relatively stable. The variation in the O_3_ concentration in South China shows an M-shaped curve, and the O_3_ concentration is higher in May and October. O_3_ pollution in Southwest China is “coming early and going fast”. The O_3_ concentration peaks around May and then falls sharply starting in June.

The monthly pattern of O_3_ can be attributed to changes in meteorological conditions and seasonal variations in precursor emissions. The decrease in the O_3_ concentration in South China in summer may be attributed to the climatic characteristic of the southwest monsoon that prevails in summer. The change in the O_3_ concentration in Southwest China is strongly affected by ultraviolet radiation. In addition, the penetration of stratospheric ozone into the troposphere is another reason supporting the high O_3_ concentration in the region.

### Weekly variation in O_3_ in China

**Figure 7:**
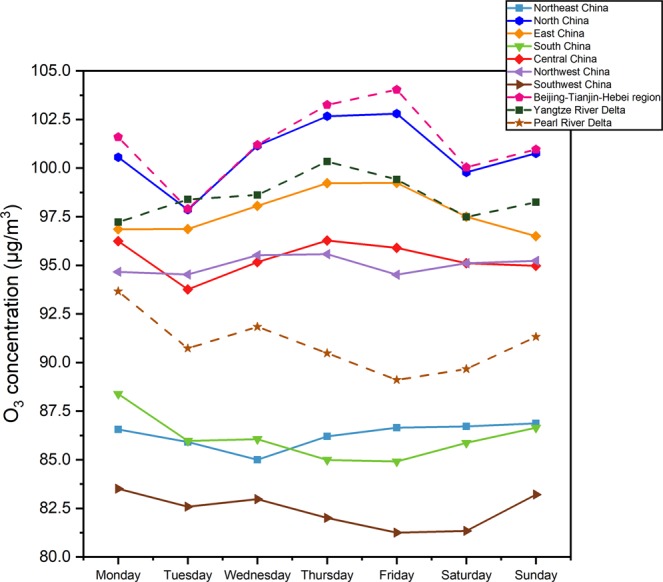
Weekly variation in the maximum daily 8-hour average concentration of O_3_ in seven geographical regions and three urban agglomerations in China during 2016-2018.

The weekly variation in the O_3_ concentration is shown in Fig. 7. The trends in different regions are not the same, but in general, they follow a W-shape. In North, Central, South, and Southwest China and in the BTH and PRD regions, the O_3_ concentration showed a valley on Tuesday. In North China, the YRD region, and the BTH region, the O_3_ concentration showed another valley on Saturday. Some scholars have studied the weekend effect of O_3_ that was first reported in New York in 1974, which suggested that the O_3_ concentration was higher during the weekend than on weekdays^44^. The weekend effect has been investigated in many other cities in the United States^45–47^, Europe^48–50^, and Asia^51–53^. The weekend effect of urban O_3_ is related to the decrease in human activities.

**Figure 8:**
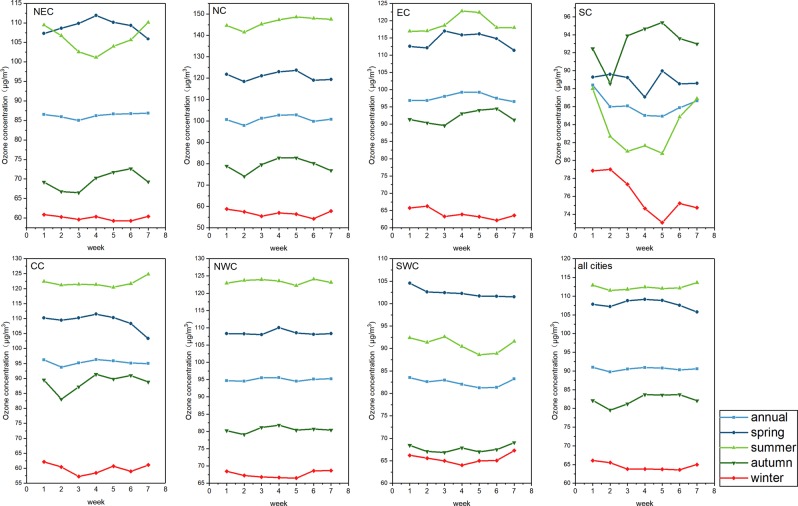
Weekly variation in the maximum daily 8-hour average concentration of O_3_ during four seasons in seven geographical regions during 2016-2018.

As shown in Fig. 8, the weekly variation in O_3_ concentration varies greatly in different regions and seasons. In our study, the valley value of O_3_ concentration often occurs on Tuesday. The weekend effect of O_3_ is more evident in the Northeast China, South China, Central China in summer, and Northwest China, Southwest China in winter to a certain degree. However, the general weekend effect of O_3_ pollution is not significant, from a national scale. The weekly variation in O_3_ concentration is affected by complex factors, the most likely of which is characteristics of urban resident activities. At present, no natural process has been found to produce climate change with a cycle of about 7 days, so Dominique *et al*.^54^ believe that the existence of such a cyclic process is manifestation of human impact on climate. Due to the obvious weekly cycle of human activities, many meteorological elements in many regions have corresponding weekly cycle characteristics^55^. Meteorological elements of some cities have been observed to have varying degrees of weekly cycle characteristics, such as temperature^56,57^, precipitation frequency^58,59^, etc., which have a significant cycle with 7-day. The change of these climate factors will further affect the generation of O_3_ in the photochemical reaction process, and thus affect the weekly variation. In general, the weekly variations in O_3_ concentration are not very prominent, which shows that the weekly changes in human activities have limited effects on O_3_ concentration.

### Daily variation in O_3_ in China

**Figure 9:**
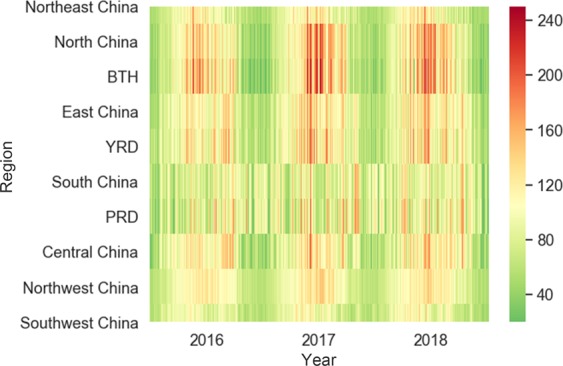
Daily variation in the maximum daily 8-hour average concentration of O_3_ in seven geographical regions and three urban agglomerations during 2016-2018. (This figure was created by using matplotlib, a Python 2D plotting library, URL:https://matplotlib.org).

Figure 9 shows the daily O_3_ concentration from January 1, 2016, to December 31, 2018. As shown in the figure, the daily variation in the O_3_ concentration is usually continuous. The change from high concentration to low concentration, or from low concentration to high concentration, is often a gradual process rather than a sudden change. In most parts of the country, the daily variation of O_3_ concentration shows an inverse U-shaped trend during each year, i.e., gradually increasing first and then decreasing. Except for South China, including the Pearl River Delta, the daily variation process of O_3_ concentration has volatility. When observing horizontally from three years, the three cycles of O_3_ variation can be clearly distinguished. We also found that for at least 1/3 of the days in the three years in each region, the O_3_ concentration exceeded the AQG, while for more than 1/3 of the days in North China, the O_3_ concentration exceeded Grade 1 of the NAAQS. When observing vertically, during the days with O_3_ pollution, the BTH, YRD, and PRD regions usually had even higher O_3_ concentrations than their neighboring areas. In short, the figure simultaneously shows the seasonal variation pattern as well as the spatial distribution characteristic of O_3_ concentration.

### Diurnal and nocturnal variation in O_3_ in China

**Figure 10:**
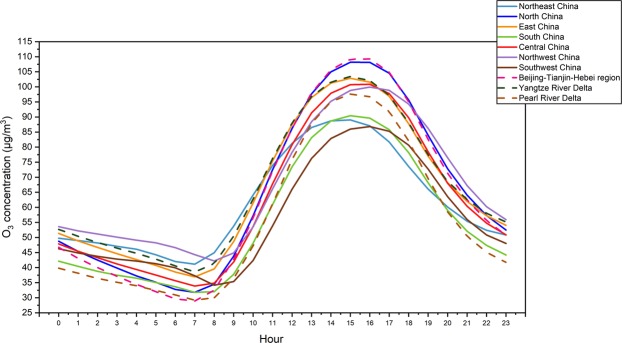
Diurnal and nocturnal variation in the average hourly concentration of O_3_ in seven geographical regions and three urban agglomerations during 2016-2018.

The hourly data on O_3_ concentration are shown in Fig. 10 and were used to investigate the diurnal and nocturnal variations in O_3_ pollutants in seven regions and three urban agglomerations in China. All regions showed a similar overall trend of O_3_ concentration, with a single peak. The O_3_ concentration was relatively lower at night, but as the sun rose, the O_3_ concentration gradually increased. The peak appeared between 14:00 and 16:00 (i.e., in the afternoon). After 16:00, the O_3_ concentration gradually decreased. The change in O_3_ concentration was affected by the temperature, solar radiation intensity, and various emissions from the surrounding environment. At night, due to the absence of solar radiation and the precursor of the photochemical reaction, the reaction was weakened and the O_3_ concentration decreased.

There are still some differences in the diurnal and nocturnal variations in the O_3_ concentration in various regions. For example, the variations in Southwest and Northwest China have a hysteresis phenomenon relative to other regions. The phenomenon is attributed to China’s vast territory, with more than 60° of east-west longitude, spanning 5,200 km and five time zones. Although Beijing time is uniformly used in China, there are actually time differences between the eastern and western regions.

### O_3_-NO_x_-VOC sensitivity regimes and influencing factors

O_3_ is a secondary pollutant, and it is mainly produced by a series of photochemical reactions among precursors. Therefore, the formation of O_3_ pollution is affected by many factors in addition to meteorological factors. The most important factors are its precursors NO_x_ and VOCs. The relationship between O_3_ and its precursor concentrations is generally nonlinear^60^. The decrease in precursor concentration does not necessarily result in a corresponding decrease in O_3_ concentration, and the sensitivity of O_3_ to NO_x_ and VOCs will be different under different environmental conditions in the same region. The O_3_-NO_x_-VOC sensitivity regimes can describe the relationship between O_3_ and its local precursors (NO_X_ and VOCs). The sensitivity relationship between O_3_ and its precursors determines the controlled types of O_3_ pollution in different regions. In brief, when the concentration of NO_x_ in the atmosphere is high, the generation of O_3_ is controlled by VOCs; however, when the VOC concentration in the atmosphere is high, O_3_ generation is controlled by NO_x_. For example, in VOC-sensitive areas, the O_3_ concentration may increase with the reduction of the NO_x_ concentration^23^. Clarifying whether O_3_ generation in a region is VOC-sensitive or NO_x-_sensitive is one of the important issues related to O_3_ generation mechanisms, which will be helpful in determining the control of targeted emissions to reduce O_3_ pollution^61^ and formulating O_3_ pollution control strategies.

In this paper, we summarize the O_3_-NO_x_-VOC sensitivity regimes in major cities in China that have been studied, and the results are shown in Supplementary Table S1. In the urban districts of most cities, including Beijing, Tianjin, Shanghai and Guangzhou, O_3_ generation is VOC-sensitive, mainly because human intervention in urban districts has greatly affected the emissions of precursors. Industry and transportation caused a large amount of NO_x_ emissions, and the titration effect suppressed the increase in the O_3_ concentration in urban areas. In these areas, the priority control of VOC emissions is more helpful in controlling local O_3_ pollution. However, in the suburban areas of some cities, such as Lanzhou, Guiyang, Chongqing, and Xuzhou, the generation of O_3_ is NO_x_-sensitive. The suburbs are less affected by anthropogenic emissions, and the migration of pollutants caused by the wind will affect O_3_ pollution in the suburbs. In these areas, to suppress O_3_ generation more effectively, priority should be given to the control of NO_x_ emissions.

In addition, the meteorological influencing factors in major cities in China are provided in Supplementary Table S1. The main meteorological factors that affect O_3_ generation include temperature, relative humidity, wind speed, wind direction, solar radiation, atmospheric pressure, cloud cover, sunshine duration, precipitation, ultraviolet radiation, visibility, and geopotential height. The statistics of their frequency are shown in Supplementary Fig. S1. In different regions, meteorological factors have heterogeneous effects on O_3_ generation. In general, O_3_ has a significant correlation with temperature and relative humidity. High temperature and low relative humidity are more conducive to the formation of O_3_, while meteorological factors such as sunshine duration, wind direction and wind speed have a crucial impact on the changes in O_3_ concentration.

Combined with the results of previous statistical analyses, we found that the O_3_ pollution affecting other cities is often caused by the synergistic effects of precursors and meteorological factors. For example, MDA8 in Beijing and its surrounding areas mainly occur at conditions of high temperature, low cloud cover, low relative humidity, weak southeast wind, low planetary boundary layer height, and the presence of a large amount of NO_x_ and VOCs^62^. In Taiyuan, when the wind direction is southerly or southwesterly, the concentration of O_3_ is higher, which indicates that the increase in O_3_ concentration in Taiyuan is not only related to the local generation but also related to the external transport from the south^63,64^. The O_3_ volume fraction and its generation rate in Langfang showed a significant positive correlation with air temperature and a significant negative correlation with total cloud cover. It is also susceptible to transmission in the southern region of Hebei and Tianjin.

From a long-term perspective, according to the characteristics of different O_3_ pollution in different regions, priority should be given to strengthen the coordinated control of the sensitive precursor emissions in the region. Forecasting in advance when meteorological conditions are adverse and taking timely NO_x_ and VOC control measures are important ways to solve regional O_3_ pollution problems.

## Conclusions

This study analyzed the spatiotemporal distributions of O_3_ concentrations in 338 prefecture-level cities in China from January 2016 to February 2019. The purpose was to understand the current status of O_3_ pollution in China with a higher spatial resolution and a longer time series. Our study has the following findings:

O_3_ had obvious spatial heterogeneity. Only a few cities met the AQG standard of the WHO. O_3_ pollution in North, East, and Central China was more serious, especially in the BTH region. The O_3_ concentrations in the BTH, YRD, and PRD regions were usually higher than those in their neighboring cities. In the spring and summer, O_3_ pollution in the north was more serious; in autumn, O_3_ pollution shifted toward the south. In winter, the O_3_ pollution problem was relatively mild across China.

O_3_ showed a significant temporal variation pattern. The O_3_ concentration increased each year from 2016 to 2018. For the monthly variation in O_3_, except for South and Southwest China, other regions showed an inverted-V curve. Although the weekly variation in O_3_ concentration was not exactly the same in different areas, some cities showed a W-shape. The O_3_ concentration was lower on Tuesday and Saturday, and no obvious weekend effect was found. The study also characterized the diurnal and nocturnal variation pattern of O_3_ concentration. The O_3_ concentration was significantly higher during the day because of factors such as solar radiation, temperature, and precursor emissions. Due to the different time zones in different cities, the western region had a remarkable lag effect compared with the eastern region.

At present, China has made some achievements in the control of PM, NOx and other pollutants; however, the problem of O_3_ pollution has become increasingly prominent. Against the background of China’s severe composite air pollution, the need for the coordinated control of multiple pollutants is becoming increasingly apparent. According to our understanding, there is coexistence of VOC control and NOx control in China’s O_3_ pollution, and the reduction of particulate matter pollution has exacerbated the problem of O_3_ pollution in China. The government should strengthen the monitoring of VOCs and combine the characteristics of O_3_ pollution in different regions to formulate more targeted O_3_ pollution control strategies to achieve a win-win situation of haze governance and O_3_ control.

## Data and methods

### The regional division of China

**Figure 11:**
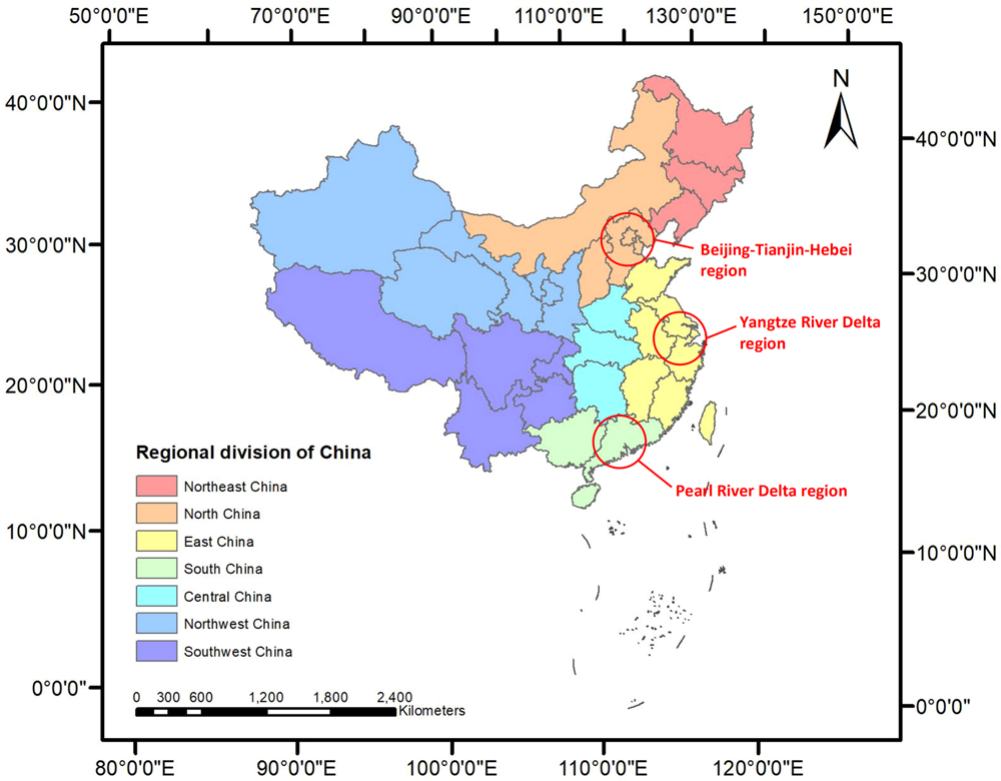
The regional division of China into seven geographical regions and three urban agglomerations. The map was generated in ArcGIS10.2, URL: http://www.esrichina-bj.cn/softwareproduct/ArcGIS/.

A total of 338 cities, including prefecture-level cities and municipalities, are used as basic study units to investigate the spatial and temporal distribution of O_3_ in China. To analyze the results more clearly, China was divided into seven geographical regions: Northeast China (NEC), North China (NC), East China (EC), Central China (CC), South China (SC), Northwest China (NWC), and Southwest China (SWC), and three urban agglomerations: Beijing-Tianjin-Hebei region (BTH), the Yangtze River Delta region (YRD), and the Pearl River Delta region (PRD) (Fig. 11).

### Ground-level O_3_ monitoring data

The China National Environmental Monitoring Center (CNEMC) continuously operates and maintains the national air quality monitoring network of China. The network has comprised 496 stations in 74 cities since 2012, and the network was extended to 1436 monitoring stations in 338 cities after 2016. The real-time concentration of O_3_ was measured by the ultraviolet absorption spectrometry method and differential optical absorption spectroscopy at each monitoring site. The instrumental operation, maintenance, data assurance and quality control were properly conducted based on the most recent revisions of China Environmental Protection Standards^2^. The real-time hourly O_3_ concentration data are continuously recorded by the CNEMC in China and are provided to the public. The data for this study were obtained during the period from 1-Jan-2016 to 28-Feb-2019.

### Maximum daily 8-hour average O_3_ and the annual average O_3_ concentration

In view of the impact of long-term O_3_ exposure on animals and plants, limits of the maximum daily 8-hour average O_3_ concentration are specified in the NAAQS. Therefore, the average hourly O_3_ concentration is calculated every 8 hours, which should include at least 6 hourly values within a given 8-hour period; otherwise, the average value is considered to be invalid. Invalid values are not accepted in subsequent analysis. Finally, the maximum daily 8-hour average O_3_ concentration in a day is used to represent the O_3_ level of that day. Additionally, the ‘technical regulation for ambient air quality assessment of China’ (on trial) (HJ 633-2013) published by the Ministry of Ecology and Environment of China (MEE) determined that the O_3_ annual assessment standard for a city is equal to the 90th percentile of MDA8.

### Statistical method

The spatial distribution of O_3_ is analyzed by calculating the average MDA8 data of all cities in each region. The annual, seasonal, monthly, weekly and daily variations in O_3_ are represented by the average of the MDA8 of each city. Diurnal and nocturnal O_3_ variation is calculated using the hourly O_3_ concentration of each city.

## Supplementary information

Supplementary information.
