# Supplementary material for: Spatiotemporal distribution of ground-level ozone in China at a city level
Source: Sci Rep. 2020 Apr 29;10:7229. doi: 10.1038/s41598-020-64111-3 (PMC7190652; doi:10.1038/s41598-020-64111-3)
Supplement: Supplementary file 1 — Supplementary information. [file 41598_2020_64111_MOESM1_ESM.pdf]

Spatiotemporal distribution of ground-level ozone in China at a city level

Guangfei Yang[1]*, Yuhong Liu[1], Xianneng Li[1]

1. Institute of System Engineering, Dalian University of Technology, Dalian, China

*Corresponding author Email:gfyang@dlut.edu.cn

Table S1. O_3-NO_x-VOC-sensitivity regimes and their meteorological influencing factors in major cities in China in relevant studies.

No.	Cities	Region	Urban agglomerations	Observation period	O3-NOx-VOC-sensitivity regimes	Meteorological factors	References
1	Harbin	NEC	/	2014-2017	/	Average temperature, relative humidity, ultraviolet radiation	Liu *et al*.(2018)[1]
2	Shenyang	NEC	/	2013-2015	/	Temperature, total radiation, wind speed	Liu *et al*.(2019)[2]
3	Dalian	NEC	/	2015.8	VOC-sensitive	Solar radiation, temperature, relative humidity, atmospheric pressure, wind speed, visibility	Bao *et al*.(2018)[3]<br>Bao *et al*.(2017)[4]
4	Beijing	NC	BTH	2009.9-2010.8	VOC-sensitive	Temperature, relative humidity, cloud cover, wind speed, solar radiation	Tang *et al*.(2012)[5]
5	Tianjin	NC	BTH	2009.8<br>2006.9-2006.10	VOC-sensitive (urban district) and NOx-sensitive (suburban)	Wind direction, solar radiation	Zhang *et al*.(2015)[6],<br>Ran *et al*.(2012)[7]<br>Han *et al*.(2011)[8]
6	Shijiazhuang	NC	BTH	2018.7	VOC-sensitive	Relative humidity, wind speed, temperature, solar radiation	Zhao *et al*.(2019)[9]
7	Taiyuan	NC	/	2013.5<br>2013-2017	VOC-sensitive	Relative humidity, wind speed, temperature	Zhang *et al*.(2015)[10]<br>Feng(2019)[11]

8	Hohhot	NC	/	2015-2016	/	Relative humidity, temperature, wind speed, distance of trajectory back, surface pressure, geopotential height	Gong *et al*.(2018)[12]
9	Langfang	NC	BTH	2014-2016	VOC-sensitive	Solar radiation, temperature, cloud cover, wind speed, wind direction	Li *et al*.(2017)[13]
10	Shanghai	EC	YRD	2017.8	VOC-sensitive (central and eastern area) and NOx-sensitive (northern and western area)	Atmospheric circulation	Luo *et al*.(2019)[14]
11	Nanjing	EC	YRD	2013.5-2013.8<br>2013.1-2016.7	VOC-sensitive	Temperature, visibility, sunshine duration, total irradiance, relative humidity, cloud cover	Shao *et al*.(2016)[15]<br>Wang *et al*.(2018)[16]
12	Hangzhou	EC	YRD	2016.7-2017.12	VOC-sensitive	Subtropical high, solar radiation, temperature, relative humidity, precipitation, wind speed	Feng *et al*.(2019)[17]
13	Ningbo	EC	YRD	2014.9-2015.8	/	Temperature, Relative humidity, wind speed, wind direction	Tong *et al*.(2017)[18]
14	Hefei	EC	YRD	2013-2015	/	Wind speed, Relative humidity, atmospheric pressure, temperature, precipitation	Zhao *et al*.(2018)[19]
15	Fuzhou	EC	/	2009-2010	/	Temperature, solar radiation, cloud cover, Relative humidity, wind speed, wind direction, atmospheric pressure, precipitation	Wang *et al*.(2011)[20]

16	Jinan	EC	/	2005.7-2005.9	/	Temperature, wind speed, sunshine duration	Yin *et al*.(2010)[21]
17	Qingdao	EC	/	2014.4	VOC-sensitive	Temperature, Relative humidity, sunshine duration	Xu *et al*.(2016)[22]
18.	Suzhou	EC	YRD	2005.4-2005.6<br>2008-2009	VOC-sensitive	Temperature, relative humidity	Xue *et al*.(2014)[23]<br>Chen *et al*.(2013)[24]
19	Jiaxing	EC	YRD	2016.8-2016.9<br>2013.8	VOC-sensitive(suburbs)	Temperature, relative humidity, wind speed	Zou *et al*.(2017)[25]<br>Shen *et al*.(2014)[26]
20	Xuzhou	EC	/	2013.8<br>2016	NOx-sensitive (suburbs)	Temperature, relative humidity, atmospheric pressure	Yan *et al*.(2016)[27]<br>Li(2018)[28]
21	Changsha	CC	/	2014.4-2015.3<br>2017.5 and 2017.9	NOx-sensitive (2017.5) and VOC-sensitive (2017.9)	Atmospheric pressure, temperature, Relative humidity	Jia *et al*.(2017)[29]<br>Fu *et al*.(2019)[30]
22	Wuhan	CC	/	2013.2-2014.10<br>2016.8	VOC-sensitive	Temperature, wind speed, relative humidity, tropical cyclones, wind direction	Lyu *et al*.(2016)[31]<br>Zeng *et al*.(2018)[32]
23	Guangzhou	SC	PRD	2015	VOC-sensitive (urban district) and NOx-sensitive (suburban)	Temperature, sunshine duration, solar radiation, atmospheric pressure, relative humidity, wind speed	Huang et al.(2018)[33]
24	Shenzhen	SC	PRD	2008.8<br>2009.8	VOC-sensitive	Temperature, wind speed	Liang *et al*.(2014)[34]<br>Yan *et al*.(2012)[35]

25	Chengdu	SWC	/	2016.9-2016.10	VOC-sensitive	Solar radiation	Tan *et al*.(2018)[36]
26	Chongqing	SWC	/	2015.8-2015.9	VOC-sensitive and NOx-sensitive (suburbs)	Solar radiation, temperature, relative humidity, wind speed, wind direction, atmospheric pressure	Su *et al*.(2018)[37] Liu *et al*.(2014)[38]
27	Guiyang	SWC	/	2016.5, 2016.8, 2016.10	VOC-sensitive (urban district) and NOx-sensitive (suburbs)	/	Fu *et al*.(2019)[39]
28	Lhasa	SWC	/	2013-2016	/	Temperature, sunshine duration, relative humidity	Wang *et al*.(2017)[40]
29	Lanzhou	NWC	/	2006.6-2006.7 2005-2015	NOx-sensitive (suburban)	Sunshine duration, atmospheric pressure	Xue *et al*.(2014)[23] Lu *et al*.(2018)[41]
30	Xi'an - Xianyang	NWC	/	2015.7 2013-2016	NOx-sensitive	Temperature, relative humidity, wind speed, wind direction	Bei *et al*.(2017)[42] Liu *et al*.(2017)[43]
31	Xining	NWC	/	2015-2017	/	Solar radiation, temperature, relative humidity, atmospheric pressure, wind speed, wind direction, precipitation	Tan *et al*.(2019)[44]
32	Yinchuan	NWC	/	2018.6	/	Temperature, relative humidity, solar radiation, ultraviolet radiation, wind speed, wind direction	Wang *et al*.(2019)[45]
33	Urumqi	NWC	/	2016.12-2017.11	/	Temperature, relative humidity, sunshine duration, wind speed, wind direction	Guan *et al*.(2019)[46]

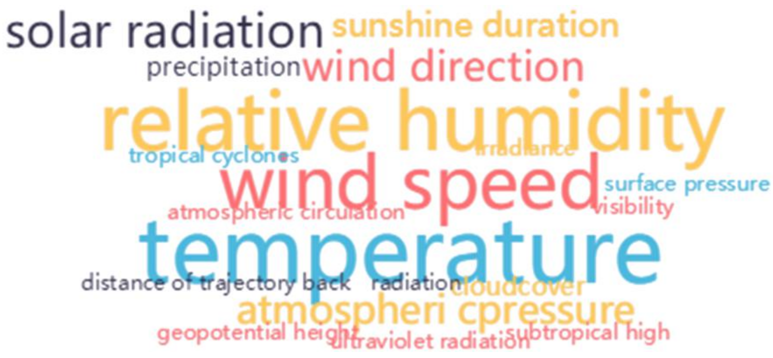

Figure S1. Word cloud diagram of frequency statistics of meteorological factors influencing O_3 generation in related literature.

References:

1.Yulian, L. *et al.* Characteristics of near surface O_3 concentration in Harbin. *Acta Scientiae Circumstantiae* **11**, 4454-4463 (2018).

2.Liu, N. *et al.* Distribution and urban – suburban differences in ground-level ozone and its precursors over Shenyang, China. *METEOROL ATMOS PHYS* **131**, 669-679 (2019).

3.Bao, Y. *et al.* Modeling of Summertime Ground-Level Ozone Pollution and Its Control Stategies in Dalian. *Environmental Monitoring in China* **34**, 9-19 (2018).

4.Bao, Y., Xu, J., Zhang, M., Tang, W. & Meng, F. The Characteristics of Ozone Pollution and Causes of A Typical Ozone Pollution Episode in Dalian. *Environmental Monitoring in China* **33**, 167-178 (2017).

5.Tang, G. *et al.* Spatial-temporal variations in surface ozone in Northern China as observed during 2009 – 2010 and possible implications for future air quality control strategies. *ATMOS CHEM PHYS* **12**, 2757-2776 (2012).

6.Zhang, M. *et al.* Sensitivity analysis of summer ozone by NCAR MM in Tianjin. *Journal of Meteorology & Environment* (2015).

7.Ran, L. *et al.* Ozone production in summer in the megacities of Tianjin and Shanghai, China: a comparative study. *ATMOS CHEM PHYS* **12**, 7531-7542 (2012).

8.Han, S. *et al.* Analysis of the Relationship between O3, NO and NO2 in Tianjin, China. *Aerosol Air Qual. Res* **11**, 128-139 (2011).

9.Zhao, L. *et al.* Pollution Characteristic and Source Apportionment of VOCs During Summer Typical Periods in Shijiazhuang. *Environmental Monitoring in China* **35**, 78-84 (2019).

10.Zhang, S., Zhang, X., Chai, F. & Xue, Z. Pollution Characteristics of NO_x, $O_3$and OX in the Ambient Air of Taiyuan City and Relativity Study. *Environmental Protection Science* **41**, 54-58 (2015).

11.Feng, X. Variation characteristics of ozone concentration in Taiyuan from 2013 to 2017. *ENVIRON CHEM* **38**, 1899-1905 (2019).

12.Gong, X., Hong, S. & Jaffe, D.A. Ozone in China: Spatial distribution and leading meteorological factors controlling O3 in 16 Chinese cities. *AEROSOL AIR QUAL RES* **18**, 2287-2300 (2018).

13.Li, L. *et al.* Influence Factors and Sensitivity of Ozone Formation in Langfang in the Summer. *Environmental Science* **38**, 4100-4107 (2017).

14.Luo, H. *et al.* Spatio-temporal variation of summertime ozone formation mechanism in Shanghai and its impact factors. *Acta Scientiae Circumstantiae* **39**, 154-168 (2019).

15.Shao, P. *et al.* Source apportionment of VOCs and the contribution to photochemical ozone formation during summer in the typical industrial area in the Yangtze River Delta, China. *ATMOS RES* **176-177**, 64-74 (2016).

16.Wang, L. *et al.* Study on the relationship between surface ozone concentrations and meteorological conditions in Nanjing, China. *Acta Scientiae Circumstantiae* **38**, 1285-1296 (2018).

17.Feng, R., Luo, K. & Fan, J. Decoding Tropospheric Ozone in Hangzhou, China: from Precursors to Sources. *ASIA-PAC J ATMOS SCI*, 1-11 (2019).

18.Tong, L. *et al.* Characteristics of surface ozone and nitrogen oxides at urban, suburban and rural sites in Ningbo, China. *ATMOS RES* **187**, 57-68 (2017).

19.Zhao, X., Dong, H., Ji, M., Cheng, L. & Geng, T. Analysis on the spatial-temporal distribution characteristics of O_3 and its influencing factors in Hefei City. *Acta Scientiae Circumstantiae* **38**, 649-660 (2018).
20.Wang, H., Lin, C., Chen, X., YU, Y. & Bai, L. Effects of weather condition on surface ozone distribution in FuZhou. *Ecology and Environmental Sciences* **20**, 1320-1325 (2011).
21.Yin, Y. *et al.* Analysis of the surface ozone during summer and autumn at a coastal site in East China. *B ENVIRON CONTAM TOX* **85**, 10-14 (2010).
22.Xu, S. *et al.* Analysis of Formation Sensitivity and Influencing Factors of Ambient O_3 in Qingdao. *The Administration and Technique of Environmental Monitoring* **28**, 19-22 (2016).
23.Xue, L.K. *et al.* Ground-level ozone in four Chinese cities: precursors, regional transport and heterogeneous processes. *ATMOS CHEM PHYS* **14**, 13175-13188 (2014).
24.Chen, K., Yang, H.B., Ma, Z.W., Bi, J. & Huang, L. Influence of temperature to the short-term effects of various ozone metrics on daily mortality in Suzhou, China. *ATMOS ENVIRON* **79**, 119-128 (2013).
25.Zou, Q. *et al.* Ozone Formation Potential and Sources Apportionment of Atmospheric VOCs during Typical Periods in Summer of Jiashan. *Environmental Monitoring in China* **33**, 91-98 (2017).
26.Shen, L. *et al.* Obervation of a Photochemical Event in Jiaxing During Summer 2013. *Environmental Science* **35**, 1662-1670 (2014).
27.Yan, R. *et al.* Establishment and application of nonlinear response surface model of ozone in the Yangtze River Delta region during summertime. *Acta Scientiae Circumstantiae* **36**, 1383-1392 (2016).
28.Li, C. Analysis on Variation Characteristics of Ozone and Correlation with Meteorological Elements in Urban Atmosphere of Xuzhou. *Environmental Science and Management* **43**, 77-81 (2018).
29.Jia, H. *et al.* Analysis of the Characteristics of Ozone Concentration in Urban Area of Changsha. *ENVIRON SCI TECHNOL* **40**, 168-173 (2017).
30.Fu, Z. *et al.* Senstivity analysis of atmospheric ozone formation to its precursors in summer of Changsha. *ENVIRON CHEM* **38**, 10-7524 (2019).
31.Lyu, X.P. *et al.* Ambient volatile organic compounds and their effect on ozone production in Wuhan, central China. *SCI TOTAL ENVIRON* **541**, 200-209 (2016).
32.Zeng, P. *et al.* Causes of ozone pollution in summer in Wuhan, Central China. *ENVIRON POLLUT* **241**, 852-861 (2018).
33.Huang, J. *et al.* Guangzhou ground level ozone concentration characteristics and associated meteorological factors. *Act Scientiae Circumstantiae* **38**, 23-31 (2018).
34.Liang, Y. *et al.* Sensitivity analysis of ozone precursor emission in Shenzhen, China. *China Environmental Science* **34**, 1390-1396 (2014).
35.Yan, M. *et al.* Ozone Pollution in Summer in Shenzhen City. *Research of Environment Sciences* **25**, 148-411 (2012).
36.Tan, Z. *et al.* Exploring ozone pollution in Chengdu, southwestern China: A case study from radical chemistry to O3-VOC-NOx sensitivity. *SCI TOTAL ENVIRON* **636**, 775-786 (2018).
37.Su, R. *et al.* Exp;oration of the formation mechanism and source attribution of ambient ozone in Chongqing with an overvation-based mode. *Science China Earth Sciences* **61**, 23-32 (2018).
38.Liu, J., Jiang, C., Song, D. & An, B. Analysis of distribution characteristics of surface ozone and its

influencing factors in summer in Chongqing. *Journal of Chongqing University* **37**, 91-98 (2014).
39.Fu, Z., Guo, J., Wang, Z., Huang, H. & Zhang, X. Analysis of atmospheric ozone formation process and the sensitivity of precursors in Guiyang. *ENVIRON CHEM* **38** (2019).
40.Wang, C., Zhang, H., Nixia, C. & Li, M. The pollution Characteristics of Surface Ozone in Lhasa-Typical City over the Tibetan Plateau. *Environmental Monitoring in China* **33**, 159-166 (2017).
41.Lu, X. *et al.* Temporal and Spatial Variation of Atmospheric Ozone in Lanzhou and Its Influencing Factors. *Earth and Environment* **46**, 355-363 (2018).
42.Bei, N., Feng, T., Wu, J. & Li, G. Simulations of summertime ozone in Xi'an and surrounding areas. *Journal of Earth Environment* **8** (2017).
43.Liu, S. *et al.* Characteristics of temporal and spatial variations of ozone and it’s influencing factor over Xi'an during 2013—2016. *Journal of Earth Environment* **8**, 541-551 (2017).
44.Tan, C. *et al.* Study on Surface Ozone Characteristics and Its Influencing Factors in Xining. *Journal of Arid Meteorology* **37**, 31-39 (2019).
45.Wang, W. *et al.* Characteristics of Typical Ozone Pollution Process Baed on Obervation Atmospheric Super Monitoring Network in Yinchuan City. *Ningxia Engineering Technology* **18**, 1-5 (2019).
46.Guan, Q., Zheng, Y., Zhao, H., Yuan, Y. & Wang, Y. Effects of PM2.5 and Meteorological Factors on Ozone in Urumqi. *Science Technology and Engineering* **19**, 275-281 (2019).
